# Acquired bone marrow aplasia and long-lasting complete remission with Loncastuximab Tesirine in a patient with transformed marginal zone lymphoma: a case report

**DOI:** 10.1007/s00277-026-07050-9

**Published:** 2026-05-11

**Authors:** Lorenzo Esposito, Ilaria Cappuccio, Fabrizio Pane, Alessandro Severino

**Affiliations:** 1 Hematology Department, Federico II University of Naples, Naples, Italy; 2 Department Clinical Medicine and Surgery, University Federico Secondo, Naples, Italy

**Keywords:** MZL, Histological transformation, Bone marrow involvement, Pancytopenia, Lonca

## Abstract

Marginal zone lymphomas (MZL) are indolent lymphomas that may undergo transformation into aggressive lymphomas, most frequently diffuse large B-cell lymphoma (DLBCL). Loncastuximab tesirine (LONCA) is an antibody–drug conjugate used in the treatment of relapsed/refractory large B-cell lymphomas. Here we present the clinical case of a patient with nodal transformed DLBCL and concomitant bone marrow involvement by indolent lymphoma successfully treated with LONCA. However the therapy was discontinued after six cycles as the patient developed treatment-related bone marrow aplasia. This case raises concerns about prolonged hematologic toxicity of LONCA, particularly in patients with pre-existing marrow involvement while highlights the potential of this drug to induce deep and durable response, even after limited exposure.

## Introduction

Marginal zone lymphomas [1] are typically characterized by an indolent clinical course. However, histological transformation (HT) into an aggressive form, typically diffuse large B-cell lymphoma, can occur in approximately 5% of patients at 5 years [2, 3]. The HT is associated with a more aggressive clinical behavior, faster progression, and a less favorable response to conventional therapies, worsening the prognosis of these patients.

Loncastuximab Tesirine (LONCA) is an antibody-drug conjugate comprising a humanized anti-CD19 monoclonal antibody conjugated to a potent pyrrolobenzodiazepine (PBD) dimer alkylating cytotoxin, SG3199, approved for the treatment of adult patients with relapsed/refractory DLBCL (R/R DLBCL) and high-grade B-cell lymphoma after two or more lines of therapy, until progression or unacceptable toxicity. In the pivotal LOTIS-2 trial [4], LONCA demonstrated significant clinical activity with a manageable safety profile. Particularly, neutropenia and thrombocytopenia are known adverse effects.

Here, we report the case of a patient with tDLBCL successfully treated with LONCA for a short period and then discontinued due to the development of a persistent pancytopenia, revealing a bone marrow aplasia.

## Case Report

In July 2020, a 66-year-old male was referred for splenomegaly and lymphadenopathy. At baseline PET/CT demonstrated increased tracer uptake in the spleen (SUV max 12.94), left common iliac region (SUV max 27.48), left internal iliac region (SUV max 4.01), and left para-rectal area (SUV max 3.85).

The patient underwent an excisional biopsy of the lesion showing the highest uptake, consistent with MZL and bone marrow biopsy performed was positive for disease infiltration, with no evidence of histological transformation at that time.

The patient received Rituximab-Bendamustine (6 cycles), achieving complete metabolic remission by December 2020, as documented by PET/CT. The bone marrow biopsy performed showed trilineage hematopoiesis, negative for lymphoma involvement.

In March 2023, he relapsed with B symptoms and conspicuous splenomegaly. A bone marrow biopsy performed at that time, demonstrated a normal cellularity with no signs of dysplasia and a slight infiltration by MZL. He was started on Ibrutinib, which provided disease control until August 2024. At that time, a new bulky cervical lymphadenopathy prompted the initiation of Zanubrutinib therapy, while awaiting lymphadenectomy.

PET/CT scan CT demonstrated increased tracer uptake in the spleen (SUV max 8.7), left common iliac region (SUV max 11.8), right cervical lymph-nodes (SUV max 10.7) and aspecific bone marrow uptake. The subsequent cervical lymph-node biopsy revealed the presence of CD19 positive large cells consistent with diagnosis of histological transformation to DLBCL In contrast, a new bone marrow biopsy confirmed involvement by CD19 positive MZL cells, with no sign of dysplasia in the remaining cell lineages.

The patient began treatment with LONCA in December 2024. At initiation, he had only mild anemia with hemoglobin 9.7 g/dL, platelets 130,000/µL, neutrophils 3,950/µL; lactate dehydrogenase was below upper limit. LONCA was administered at 0.15 mg/kg every 21 days for the first two cycles, followed by 0.075 mg/kg for subsequent cycles, as per institutional protocol and regulatory indication.

After six cycles, in April 2025, the patient developed a trilinear cytopenia with severe neutropenia (hemoglobin 10.6 g/dL, platelets 52,000/µL, neutrophils 330/µL). LONCA was discontinued Subsequent PET/CT confirmed the complete metabolic remission and bone marrow aspirate showed no evidence of lymphoma cells. The patient was placed on close follow-up while awaiting recovery from pancytopenia.

Three months following the end of LONCA, the patient remained in complete remission; however, pancytopenia with reticulocytopenia persisted, prompting a bone marrow biopsy. The histological examination showed a variable cellularity, up to a maximum of 10%, without evidence of lymphoma infiltration. Flow cytometry, karyotype analysis and Next Generation Sequencing performed on the bone marrow aspirate excluded abnormalities consistent with myelodysplasia. Paroxysmal Nocturnal Hemoglobinuria testing was negative, and vitamin deficiencies or viral infections, such as CMV and parvovirus B19, were ruled out leading to acquired bone marrow aplasia diagnosis [5].

At the most recent follow-up in December 2025 (eight months after LONCA discontinuation), the patient was still in complete remission, and no further lymphoma-directed treatment had been initiated. Blood counts are only slightly improving with hemoglobin 10.8 g/dL, platelets 58,000/µL and neutrophils 1,430 µ/L.

## Discussion

Marginal zone lymphomas are typically indolent B-cell neoplasms that may undergo histological transformation into aggressive lymphomas, most commonly DLBCL, a process associated with poorer prognosis and the need for more intensive treatment strategies.

Loncastuximab tesirine is an antibody–drug conjugate targeting CD19, delivering a highly potent pyrrolobenzodiazepine (PBD) dimer cytotoxin to malignant B cells. Its efficacy in relapsed/refractory DLBCL, including transformed cases, has been demonstrated in clinical trials. However, hematologic toxicity is generally reported as manageable and reversible.

In this context, our case presents a clinically relevant deviation from the expected safety profile, developing severe and persistent pancytopenia, ultimately evolving into bone marrow aplasia.

The prolonged nature of cytopenia observed in this case is unusual and cannot be fully explained by the known toxicity profile of loncastuximab tesirine alone. Several factors may have contributed to this outcome. First, the PBD payload of loncastuximab tesirine, due to its high cytotoxic potency and lipophilicity [6], may exert off-target effects on the bone marrow microenvironment, including stromal components essential for hematopoietic recovery. The presence of bone marrow involvement by indolent lymphoma may have increased PBD concentration in the hematopoietic niche. Second, prior exposure to bendamustine, a drug known to induce long-lasting immunosuppression and stem cell damage, may have played a role through cumulative or delayed toxicity, despite in our case the bone marrow biopsy performed three years after bendamustine treatment showed normal cellularity.

Importantly, extensive diagnostic work-up excluded alternative causes of bone marrow failure. No evidence of residual lymphoma infiltration was detected, and both flow cytometry and next-generation sequencing analyses did not support a diagnosis of myelodysplastic syndrome. In addition, infectious causes and paroxysmal nocturnal hemoglobinuria were ruled out. While causality cannot be definitively established, the temporal association with loncastuximab exposure and the exclusion of other etiologies strongly support a treatment-related effect.

Another aspect of this case is the achievement complete metabolic remission and eradication of bone marrow disease, suggesting activity across both aggressive and indolent components. This observation is in line with the biological rationale of CD19 targeting and parallels the activity observed with other CD19-directed therapies, such as CAR-T cells, which have shown efficacy in indolent lymphomas, including MZL. Furthermore, the response persisted despite early treatment discontinuation, supporting the notion that LONCA may induce deep, durable responses even when cumulative exposure is limited.

Overall, this case underscores the importance of close hematologic monitoring during treatment with LONCA, particularly in individuals with prior marrow damage or exposure to stem cell-toxic agents. Further studies are needed to better characterize the risk factors for prolonged cytopenia and to optimize treatment strategies in this setting.

## Conclusions

In conclusion, we report a case of prolonged bone marrow aplasia following treatment with loncastuximab tesirine in a patient with transformed lymphoma and pre-existing bone marrow involvement.

Although a direct causal relationship cannot be definitively established, this observation raises awareness of a potential severe and persistent hematologic toxicity associated with this agent, particularly in heavily pretreated patients or those with bone marrow infiltration.

These findings highlight the need for careful monitoring and further investigation into predictive factors of toxicity to better guide clinical decision-making.

## Data Availability

No datasets were generated or analysed during the current study.
